# Dapoxetine prevents neuronal damage and improves functional outcomes in a model of ischemic stroke through the modulation of inflammation and oxidative stress

**DOI:** 10.1007/s00210-023-02601-7

**Published:** 2023-07-07

**Authors:** Sarah Sameh Abdel-Hameed, Mahmoud El-Daly, Al-Shaimaa F. Ahmed, Amany A. Bekhit, Gehan H. Heeba

**Affiliations:** 1 Department of Pharmacology and Toxicology, Faculty of Pharmacy, Minia University, Minia, Egypt; 2 Department of Biochemistry, Faculty of Pharmacy, Minia University, Minia, Egypt

**Keywords:** Dapoxetine, Cerebral ischemia, Oxidative stress, Apoptosis, Caspase-3, NO

## Abstract

Stroke is a medical emergency that is associated with substantial mortality and functional disability in adults. The most popular class of antidepressants, selective serotonin reuptake inhibitors SSRIs, have recently been shown in studies to have positive effects on post-stroke motor and cognitive function. Thus, we hypothesized that dapoxetine (DAP), a short-acting SSRI, would be effective against cerebral ischemia/reperfusion injury. Adult male Wister rats (200–250 g) were subjected to a sham operation or bilateral common carotid artery occlusion (BCCAO) for 30 min followed by 24 h of reperfusion to induce global cerebral ischemia/reperfusion (I/R) injury. Rats were treated with vehicle or DAP (30 or 60 mg/kg, i.p.) 1 h before BCCAO. The neurobehavioral performance of rats was assessed. The infarct volume, histopathological changes, oxidative stress parameters, and apoptotic and inflammatory mediators were determined in the brain tissues of euthanized rats. Our results confirmed that DAP significantly ameliorated cerebral I/R-induced neurobehavioral deficits, reduced cerebral infarct volume, and histopathological damage. Moreover, DAP pretreatment reduced lipid peroxidation, caspase-3, and inflammatory mediators (TNF-α and iNOS) compared to I/R-injured rats. Thus, DAP pretreatment potentially improves neurological function, and cerebral damage in cerebral ischemic rats may be partly related to the reduction in the inflammatory response, preservation of oxidative balance, and suppression of cell apoptosis in brain tissues.

## Introduction

Stroke is one of the cerebrovascular illnesses associated with a high mortality rate. It is the fifth leading cause of death globally (Flynn et al. 2008). A substantial restriction in cerebral blood flow occurs, preventing essential nutrients and oxygen from reaching the brain and resulting in neuronal dysfunction (Simats et al. 2016). This condition causes physical limitations as well as severe cognitive impairment, which necessitates immediate medical attention (W. Li et al. 2015). Unfortunately, an early reperfusion therapy to improve blood flow might exacerbate the damage, resulting in a syndrome known as cerebral ischemia/reperfusion injury (CIRI) (Hu et al. 2015). Therefore, the development of novel therapeutic approaches aiming to restore cerebral blood flow without further cerebral injury should limit the severity of post-ischemic cerebral damage and enhance overall functional outcomes.

The stranglehold of glucose and oxygen supplies during an ischemic stroke attack generates an excessive amount of reactive oxygen species (ROS), glutamate accumulation, and influx of calcium, which initiates cytochrome C secreted by mitochondria, and caspases are activated and promote apoptotic cell death (Dirnagl et al. 1999; Shan et al. 2020). The antioxidant capacity of tissues is the key endogenous defense mechanism against ROS damage (Islekel et al. 1999). Once the antioxidant system is disrupted, oxidative damage occurs to brain lipids, proteins, and DNA, which contributes to neuronal cell death and brain dysfunction (Guo et al. 2017). Plus, CIRI stimulates the synthesis of neuroinflammatory cytokines, which activates numerous pathways that contribute to the destruction of cerebral tissue. Neuroinflammation and oxidative stress both play a significant role in CIRI (Jurcau & Simion 2021). Numerous neuroprotective medicines have been proposed in preclinical investigations, but none have proven effective in actual clinical settings (Horn et al. 2001; Savitz et al. 2017; Serebruany 2006; S. Y. Xu & Pan 2013; Zhang et al. 2007). Selective serotonin reuptake inhibitors (SSRIs) are frequently used for psychiatric disorders such as anxiety and depression (Vaswani et al. 2003). This class of drugs can stimulate hippocampus neurogenesis and have powerful protective effects against ischemia-hypoxia (Schmidt & Duman 2007)**.** Besides, there is strong evidence that SSRIs improved functional recovery after stroke through the reduction of inflammation and the enhancement of neurogenesis (Chollet et al. 2018). Several studies demonstrated the effectiveness of different SSRIs like fluoxetine, sertraline (Jolkkonen et al. 2000; Shin et al. 2009), and paroxetine (Naderi et al. 2018) in animal models of cerebral ischemia. For example, Buga et al. (2016) reported that fluoxetine reduced the expression of HTR 2B mRNA which in turn stimulated neurogenesis in the subventricular zone and improved animal behavioral outcomes.

Dapoxetine (DAP) is a short-acting member of the SSRIs and uses a mechanism of action comparable to current SSRIs (Andersson et al. 2006). DAP binds to serotonin, dopamine, and norepinephrine reuptake transporters and inhibits serotonin, dopamine, and norepinephrine uptake with an order of potency: serotonin > norepinephrine ≫  ≫ dopamine (Kendirci et al. 2007; Chris G McMahon 2012ab). Primarily, DAP is the first oral on-demand agent approved for the treatment of premature ejaculation (McMahon et al. 2006). A previous study found that DAP potently controls cloned Kv4.3 potassium voltage-gated channels, which are responsible for controlling the release of neurotransmitters, and provides more understanding of the mechanism underlying some of the positive efficacy of this medication (Clement et al. 2007; C. G. McMahon 2012ab).

Despite the fact that the major clinical indication for selective serotonin reuptake inhibitors (SSRIs) is the treatment of depression (Galecki et al. 2018), multiple studies had proven their effectiveness in neurodegenerative illnesses that commonly follow cerebral ischemia injury (Gaur & Kumar 2010; F. Xu et al. 2019). The SSRI-mediated increase in brain-derived neurotrophic factors and activation of neurogenesis might explain their neuroprotective effects in stroke (Do Hoon Kim et al. 2007ab). A recent report illustrated the neuroprotective effects of DAP against glutamate-induced cell death through mitochondrial depolarization in cultured primary rat hippocampal neurons (Jeong et al. 2017). On the other hand, the possible in vivo neuroprotection by DAP against stroke has not been fully elucidated. The current study intends to investigate the therapeutic potential of dapoxetine in experimental animals subjected to global cerebral ischemia/reperfusion injury by comparing its two dosages (60 mg against 30 mg).

## Materials and methods

### Animals

Adult male Wistar rats weighing 250–300 g were obtained from the animal care unit of Nahda University in Beni Suef (NUB), Beni Suef, Egypt. All rats were housed for 2 weeks as an acclimatization period before the experiment. Rats were exposed to a dark/light cycle of 12/12 h. Standard commercial rat chow and tap water were available for all animals during the experimentation period. All experimental procedures were approved by the Commission on Ethics of Scientific Research, Faculty of Pharmacy, Minia University, Egypt (Approval Number: ES29/2020).

### Chemicals

Dapoxetine was a gift from Rameda Pharmaceuticals (6^th^ of October City, Giza, Egypt). 2,3,5-Triphenyltetrazolium chloride (TTC) stain was purchased from Sigma-Aldrich Chemical (USA). Assay kits for the determination of total protein, malondialdehyde (MDA), reduced glutathione (GSH), and catalase activity were purchased from Biodiagnostic (Egypt). Bradford protein assay kit (SK3041) was provided by Bio Basic Inc. (Markham, Ontario, L3R8T4, Canada). TGX Stain-Free™ FastCast™ Acrylamide Kit (SDS-PAGE) was provided by Bio-Rad Laboratories Inc.

(Cat. Number: 161–0181). Primary antibodies against transforming growth factor receptor 2 (TGF-β RII) (Cat. Number: SC-17791), tumor necrosis factor-α (TNF-α) (Cat. Number: SC-52746), inducible nitric oxide synthase (iNOS) (Cat. Number: SC-7271), and caspase-3 (Cat. Number: SC-56053) were purchased from Santa Cruz.

### Experimental groups

Dapoxetine was administered in the current study at doses of 30 mg/kg and 60 mg/kg based on previously published data (Qin et al. 2017) and preliminary results from our lab. Forty animals were randomly divided into the following five groups (each consisting of 8 animals):

1. Sham-operated group: rats were subjected to a sham operation and received the vehicle only.
2. Sham-operated dapoxetine-pretreated group: rats were injected intraperitoneally with dapoxetine (60 mg/kg) 1 h before surgery.
3. Untreated cerebral I/R group: rats were exposed to global cerebral ischemia for 30 min followed by 24 h of perfusion.
4. Thirty milligrams/kilogram dapoxetine–pretreated group: rats received dapoxetine (30 mg/kg, i.p.) 1 h before induction of cerebral I/R injury.
5. Sixty milligrams/kilogram dapoxetine–pretreated group: rats received dapoxetine (60 mg/kg, i.p.) 1 h before induction of cerebral I/R injury.

Sham groups were exposed to the same conditions of surgery except clamping of carotid arteries.

### Induction of global ischemia/reperfusion injury

Bilateral common carotid artery occlusion (BCCAO) procedure was used for the induction of transient global cerebral ischemia (Seif-el-Nasr & Fahim 2001). Briefly, rats received an i.p. injection of ketamine (80 mg/kg) and xylazine (10 mg/kg) for induction of anesthesia and were placed in a supine position on a thermostatically controlled heating pad. A midline incision in the neck was made, and common carotid arteries were exposed under a dissecting microscope. Careful blunt dissection was applied to free carotid arteries from adventitial sheets and vagus nerves. Induction of global cerebral ischemia was achieved by the application of non-traumatic aneurysm micro-artery clamps on both common carotid arteries. Cessation of blood flow was verified visually by blanching the artery segments after the occlusion site. After maintaining ischemia for 30 min, the clamps were removed, and the wound was properly sutured to allow reperfusion for 24 h.

### Assessment of neurobehavioral performance

After the 24-h reperfusion phase, all animals were subjected to neurobehavioral testing to evaluate their motor and exploratory activities as well as sensorimotor functions. These tests include open-field, closed-field, beam-walking, and adhesive-removal tests.

#### Open-field test

The open-field test evaluates motor and exploratory activities after cerebral ischemia. The open field used in the current study consists of a wooden box (60 × 60 × 30 cm) with a white floor painted with black lines to form 16 equal squares (15 × 15 cm). Each rat was introduced into the open field for 5 min, while a video camera recorded its activity (Shehata et al. 2020). The number of squares the animal crossed during the 5-min period was registered as the ambulation frequency. Similarly, rearing (number of times the animal stood on hind limbs) and grooming (number of times the animal cleaned itself) frequencies were recorded.

#### Closed-field test

Spontaneous motor activity for each rat was assessed in a photoactometer activity cage (Letica®, Spain). When the animal crosses any of the continuous beams of light by its movement, the corresponding photoelectric cells are activated. The number of crossings is counted automatically for a period of 10 min for each animal (Khan et al. 2020).

#### Beam-walking test

The beam-walking test discovers any locomotor deficits associated with cerebral ischemia. It was applied in the current experiment to assess coordination, balance, and integration of motor movements. The used equipment consists of a white plastic beam (2 cm wide and 100 cm long) positioned at a height of 65 cm from the lab bench by 2 supporting legs. A black box (25 × 25 × 20.5 cm) was installed on one end of the beam so that it is open toward the beam. Rats were trained five times daily for 3 consecutive days before the experiment day. The test begins when the animal is positioned on the beam end opposite the black box. Once the rat entered the safe black box, it was allowed to rest there for about 15 s before the next trial. The performance of each rat was recorded at the end of the reperfusion period and rated on a 0–6-point scheme with a help of a slow-motion camera (B. Lin, Levy, Raval, Perez-Pinzon, & Defazio, 2010). Scoring of each animal was carried out according to the following scheme:

- 0: the rat was unable to stay on the beam.
- 1: the rat was able only to stay on the beam.
- 2: the rat tried to traverse the beam but fell.
- 3: the rat traversed the beam with more than 50% hind-limb foot slips.
- 4: the rat traversed the beam with fewer than 50% foot slips.
- 5: the rat traversed the beam with only one slip.
- 6: the rat traversed the beam with no slips.

#### Adhesive-removal test

The adhesive-removal test evaluates sensorimotor deficits induced by cerebral ischemia. Rats were trained five times per day for 3 consecutive days before the experiment day. Two small pieces of adhesive tapes (1 × 1.25 cm) were applied to both forepaws, and the time from the attachment of adhesive tapes until the rat completely removed them using its teeth was recorded on each of three trials lasting up to 180 s (Q. M. Lin et al. 2013). The average time required for complete tape removal was recorded for each rat on the day of surgery and after 24 h of reperfusion.

### Sample collection and tissue preparation

Rats were humanely decapitated under mild anesthesia for 24 h, after the reperfusion phase and the performance of all behavioral tests. The brains of each group were harvested and divided into two sets. The first set of brains from each group was isolated after carefully peeling off the skull bones, rinsed with ice-cold isotonic saline, frozen for 45 min, and used for the measurement of cerebral infarct volume. The brains of the second set were removed following the same procedure after intra-cardiac whole-body perfusion by freshly prepared cold saline (0.9% NaCl) and divided longitudinally into two halves. For each rat, one half was rapidly frozen in liquid nitrogen and stored at − 20 °C and later used for biochemical assays and western blotting analyses. The other half was fixed in neutrally buffered 10% formalin and used for histopathological evaluation.

### Measurement of cerebral infarct volume

The whole brain was frozen for 45 min before being carefully sliced into 2 mm thick serial coronal pieces. As described previously (Joshi et al. 2004), brain slices were immersed in phosphate-buffered saline (PBS) containing 0.05% TTC and incubated for 30 min at 37 °C in the dark. After staining, the brain slices were washed 3 times in PBS, each for 1 min. The viable metabolically active tissues can absorb the water-soluble TTC dye and convert it into an insoluble form intracellularly, hence stained dark red, while the infarcted areas remain unstained and appear as white areas. A computerized image analysis software program (ImageJ, NIH, USA) was used to measure the infarct area in each scanned brain slice, which was multiplied by its thickness to calculate the infarct volume (Heeba & El-Hanafy 2012).

### Measurement of oxidative stress parameters

Homogenates of brain tissues (20% w/v) were prepared in cold phosphate buffer saline solution (PBS) (0.05 M, pH 7.4) using a motor-driven homogenizer (LabGEN 7, Cole-Parmer, USA). The tissue homogenates were immediately centrifuged for 15 min at 4000 rpm at 4 °C. The supernatants were separated and used for the estimation of brain levels of MDA, reduced GSH, and catalase activity. All assays were carried out calorimetrically using commercially available kits according to the manufacturer’s instructions (Biodiagnostic, Egypt).

### Western blot analysis

The expression levels of TGF-β RII, caspase-3, iNOS, and TNF-α proteins in brain tissues were evaluated by SDS-PAGE followed by western blotting (immunoblotting). The total protein concentration in each sample was determined using a Bradford assay kit according to the manufacturer’s instructions to ensure equal loading. The supernatants were mixed with an equal volume of 2 × Laemmli sample buffer and boiled at 95 °C for 5 min to ensure complete denaturation of the proteins. A total amount of approximately 20 μg protein from each sample was loaded onto the wells of the polyacrylamide gel. The proteins were separated on a polyacrylamide gel according to their molecular weight, and SDS-PAGE TGX Stain-Free FastCast® was prepared according to the manufacturer’s instructions. Protein bands were transferred from the developed gel to a membrane in the transfer tank with a 1 × transfer buffer. The blot was run for 7 min at 25 V using the Bio-Rad Trans-Blot Turbo system. The membrane was washed once in Tris-buffered saline with Tween 20 (TBST) and then blocked using 3% bovine serum albumin (BSA) prepared in the same buffer for 1 h at room temperature. The membrane was incubated overnight with any of the primary antibodies (diluted according to the manufacturer’s recommendations) at 4 °C. The blot was washed three times in TBST, then incubated with the HRP-conjugated secondary antibody (goat anti-rabbit IgG-HRP mAb, Novus Biologicals) solution for 1 h at room temperature. The blots were developed with a chemiluminescent reagent, and the chemiluminescence signals were captured with a CCD camera–based imager. Image analysis software was used to read the band intensity of the target proteins, which was normalized against beta-actin (a housekeeping protein) by protein normalization on the ChemiDoc MP imager (Shehata et al. 2020).

### Histopathological examination of brain tissues

Formalin-fixed brain samples were dehydrated by passing through ascending concentrations of ethyl alcohol. Dehydrated samples were cleared in xylene and embedded in paraffin blocks. Coronal sections of 5 μm thickness were stained with hematoxylin–eosin (H&E) and then subjected to semi-qualitative microscopical analysis under a light microscope (OlympusCX41). The method described previously (Kato et al. 1991) was adopted to score the cerebral histopathological changes as (0) for no observed damage, ( +) > 25% of the area is damaged, (+ +) 25–50% of the area is damaged, (+ + +) > 75% of the area is damaged, and (+ +  + +) < 75% of the area is damaged.

### Statistical analysis

Data are expressed as mean ± standard error of the mean (SEM) and were analyzed for statistically significant differences using one-way analysis of variance (ANOVA) followed by the Tukey–Kramer post-analysis test to compare all groups. The beam-walking test scores were analyzed for statistically significant differences using the Kruskal–Wallis test followed by Dunn’s multiple comparison test. Differences between groups were statistically significant at *p* values < 0.05. GraphPad Prism® (Version 7.00 Windows) was used for statistical calculations.

## Result

### Performance of rats

#### Open-field test

**Fig. 1:**
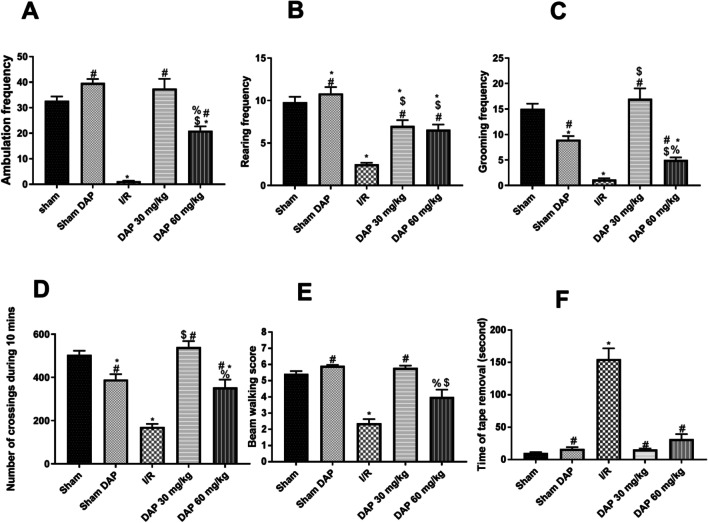
Bar charts showing the effect of the global cerebral ischemia/reperfusion model on the ambulation frequency (**A**), rearing frequency (**B**) and grooming frequency (**C**) as a part of the open-field test, number of crossings in 10 min (**D**), beam-walking test score (**E**), and the time of tape removal (seconds) (**F**) and their alteration on the sham-operated group, the sham dapoxetine-pretreated group, the 30 mg/kg dapoxetine–pretreated group, and the 60 mg/kg dapoxetine–pretreated group. Data are represented as mean ± S.E.M. #Significantly different from the I/R group at *p* < 0.05. *Significantly different from the sham group at *p* < 0.05. $Significantly different from the sham dapoxetine group at *p* < 0.05. %Significantly different from the 30 mg/kg dapoxetine–pretreated group at *p* < 0.05. I/R cerebral ischemia/reperfusion, DAP dapoxetine

When rats were subjected to ischemia injury for 30 min followed by reperfusion for 24 h, they developed substantial locomotor deficits. I/R rats displayed a significant reduction (*p* < 0.05) of ambulation, rearing, and grooming frequencies in comparison with the sham-operated group as illustrated in Fig. 1A–C, respectively. Contrarily, rats pretreated with DAP at 30 mg/kg significantly improved the motor and exploratory activity parameters to reach the levels observed in sham-operated rats (Fig. 1A–C). Although the higher dose of DAP 60 mg/kg significantly increased the frequencies of ambulation, rearing, and grooming when compared to I/R rats, it did not improve ambulation and grooming frequencies like the smaller dose.

#### Closed-field test

Data in Fig. 1D demonstrate that induction of global cerebral ischemia/reperfusion significantly (*p* < 0.05) reduced the 10-min motor activity in untreated I/R rats in comparison with sham-operated rats and sham DAP-pretreated rats. On the other hand, administration of DAP at the dose of 30 mg/kg resulted in significant (*p* < 0.05) improvement in motor activity compared to the untreated I/R group. Pretreatment with 60 mg/kg DAP also improved the motor activity compared to untreated I/R animals. However, this improvement was less than that was observed with a 30 mg/kg dose.

#### Beam-walking test

Data in Fig. 1E revealed that the beam-walking test score of ischemic rats was significantly reduced (*p* < 0.05) in comparison with sham controls. Interestingly, prior administration of DAP 30 mg/kg caused complete normalization of this test score (5.80 ± 0.13 vs. 5.42 ± 0.18 in sham rats). Nevertheless, the high dose of DAP (60 mg/kg) significantly improved the movement coordination over the I/R group, but the test score was still significantly lower than the 30 mg/kg DAP–pretreated group (Fig. 1E).

#### Adhesive-removal test

Ischemic rats required a longer duration (*p* < 0.05) for complete adhesive tape removal in comparison with sham-operated rats (Fig. 1F), which indicates deteriorated somatosensory function in these animals. While the administration of DAP before I/R injury improved the somatosensory function significantly (*p* < 0.05), which was manifested as the reduced time of tape removal compared to the I/R group (Fig. 1F).

### Cerebral infarct volume

**Fig. 2:**
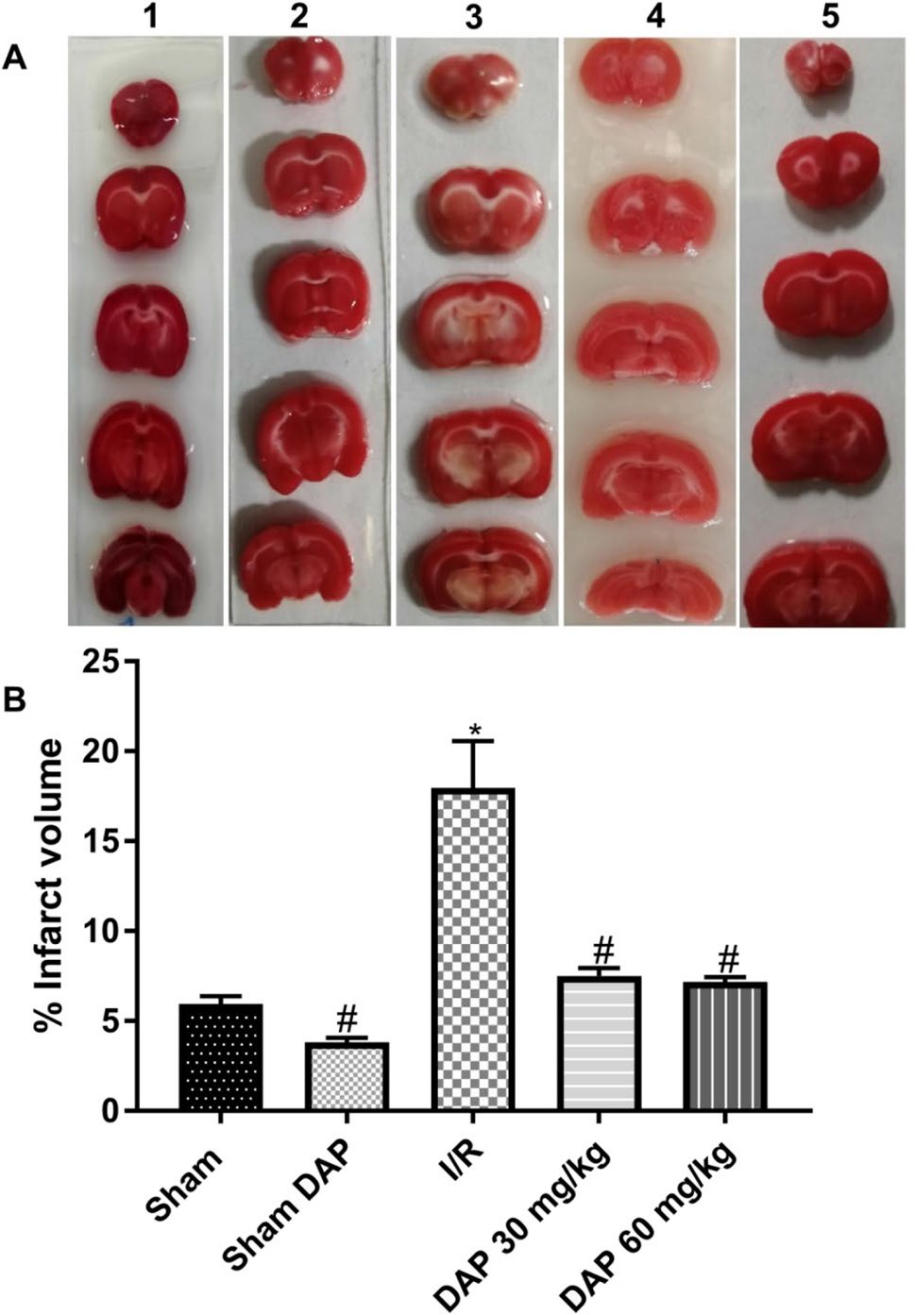
**A** Representative photographs of coronal brain sections of the sham-operated group (1), the sham dapoxetine-pretreated group (2), the untreated I/R group (3), the 30 mg/kg dapoxetine–pretreated group (4), and the 60 mg/kg dapoxetine–pretreated group (5). Dark red–colored regions in 0.05% TTC-stained sections indicate the living areas, while infarcted areas are unstained and appear white, and **B** bar chart showing a semi-quantitative analysis of cerebral infarct volume of the sham-operated group, the sham dapoxetine-pretreated group, the untreated I/R group, the 30 mg/kg dapoxetine–pretreated group, and the 60 mg/kg dapoxetine–pretreated group. (The infarct volume is represented as a percentage.) Data are represented as mean ± S.E.M. #Significantly different from the I/R group at *p* < 0.05. *Significantly different from the sham group at *p* < 0.05. I/R cerebral ischemia/reperfusion, DAP dapoxetine

According to the data in Fig. 2, I/R injury produced a significant infarction in the brain of I/R rats when compared to sham-operated rats. Furthermore, our data demonstrated that prior administration of DAP at two dose levels (30 or 60 mg/kg) has neuroprotective properties against I/R-induced brain infarction. Obviously, DAP normalized the infarct volume to the level observed in the sham-operated group (Fig. 2).

### Effect on oxidative stress parameters (lipid peroxidation, reduced glutathione, and catalase) in brain tissues

As illustrated in Table 1, I/R injury increased brain levels of oxidative stress markers in comparison with sham-operated rats. For I/R rats, the result showed an approximate two-fold increase in lipid peroxidation levels (MDA) over sham-operated rats. Besides, these rats demonstrated limited endogenous antioxidant capacity indicated by low levels of reduced glutathione (GSH) and catalase activity. In contrast, the pretreatment with DAP in two doses (30, 60 mg/kg) used in our experiments was successful in suppressing the elevated level of MDA in I/R rats. Similarly, Table 1 further demonstrated the antioxidant effects of the pre-administration of DAP 30 mg/kg via normalization of the GSH level. Yet GSH level in the group that received DAP 60 mg/kg prior to I/R injury remained unchanged. Surprisingly, neither of the two DAP doses (30, 60 mg/kg) reversed I/R-induced decreases in cerebral catalase activity (Table 1).

**Table 1:** Effect of the global cerebral ischemia/reperfusion model on cerebral levels of MDA and GSH and cerebral catalase activity and their alteration by different doses of dapoxetine

	Sham	Sham DAP	I/R	I/R + DAP	
				30 mg/kg	60 mg/kg
MDA (nmol/mg protein)	4.39 ± 0.63	3.89 ± 0.78#	9.19 ± 1.47*	3.69 ± 0.64#	2.65 ± 0.86#
GSH (nmol/mg protein)	0.74 ± 0.03	0.60 ± 0.07	0.49 ± 0.02*	0.68 ± 0.03#	0.36 ± 0.05*%
Catalase (U/mg protein)	7.63 ± 0.76	7.08 ± 1.06	2.53 ± 0.31*$	3.33 ± 0.24*$	2.26 ± 0.33*$

Data are represented as mean ± S.E.M

*I/R* cerebral ischemia/reperfusion, *DAP* dapoxetine

* Significantly different from the sham group at *p* < 0.05

# Significantly different from the I/R group at *p* < 0.05

$ significantly different from the sham dapoxetine group at *p* < 0.05

%Significantly different from 30 mg/kg dapoxetine–pretreated group at *p* < 0.05

### Effect on cerebral protein expression of TNF-α, Caspase-3, TGF-β RII, and iNOS

**Fig. 3:**
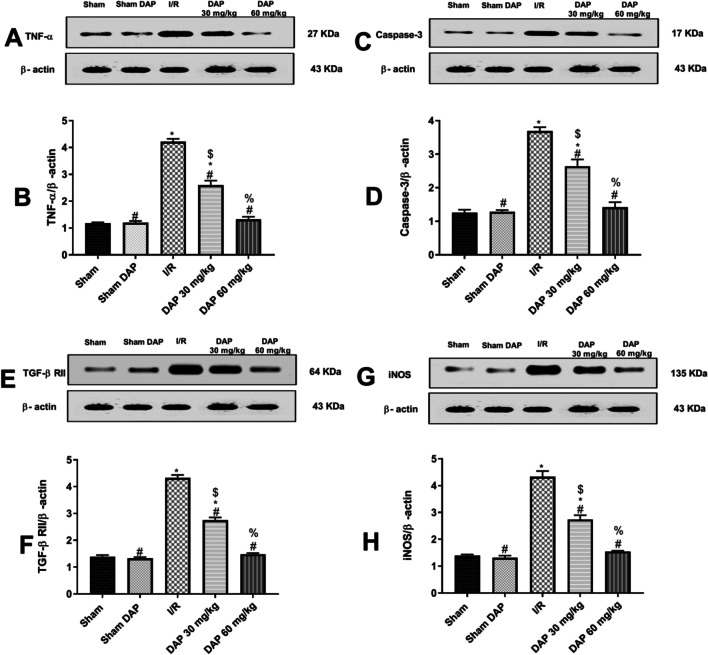
Western blot bands showing cerebral expression of TNF-α and β-actin (**A**), caspase-3 and β -actin (**C**), TGF- β RII and β -actin (**E**), and iNOS and β -actin (**G**) and bar charts showing quantitative analysis of cerebral TNF-α (**B**), cerebral caspase-3 (**D**), cerebral TGF- β RII (**F**), and cerebral iNOS (**H**) expression on the sham-operated group, the sham dapoxetine-pretreated group, the untreated I/R group, the 30 mg/kg dapoxetine–pretreated group, and the 60 mg/kg dapoxetine–pretreated group. Data are represented as mean ± S.E.M. #Significantly different from the I/R group at *p* < 0.05. *Significantly different from the sham group at *p* < 0.05. $Significantly different from the sham dapoxetine group at *p* < 0.05. %Significantly different from the 30 mg/kg dapoxetine–pretreated group at *p* < 0.05. I/R cerebral ischemia/reperfusion, DAP dapoxetine

I/R injury provoked a significant (*p* < 0.05) elevation in the cerebral protein expression of TNF-α (Fig. 3A, B) and caspase-3 (Fig. 3C, D) in I/R rats. Pretreatment with DAP, on the other hand, drastically reduced the expression of these proteins in a dose-dependent manner when compared to the I/R group. Likewise, cerebral I/R injury greatly enhanced the protein expression of TGF-β RII (Fig. 3E, F) and iNOS (Fig. 3G, H), which was dropped by prior administration of DAP in a dose-dependent manner. Nonetheless, animals pretreated with a high dose of DAP (60 mg/kg) considerably adjusted these inflammatory and pro-apoptotic parameters.

### Effect on histopathology of brain tissues

**Fig. 4:**
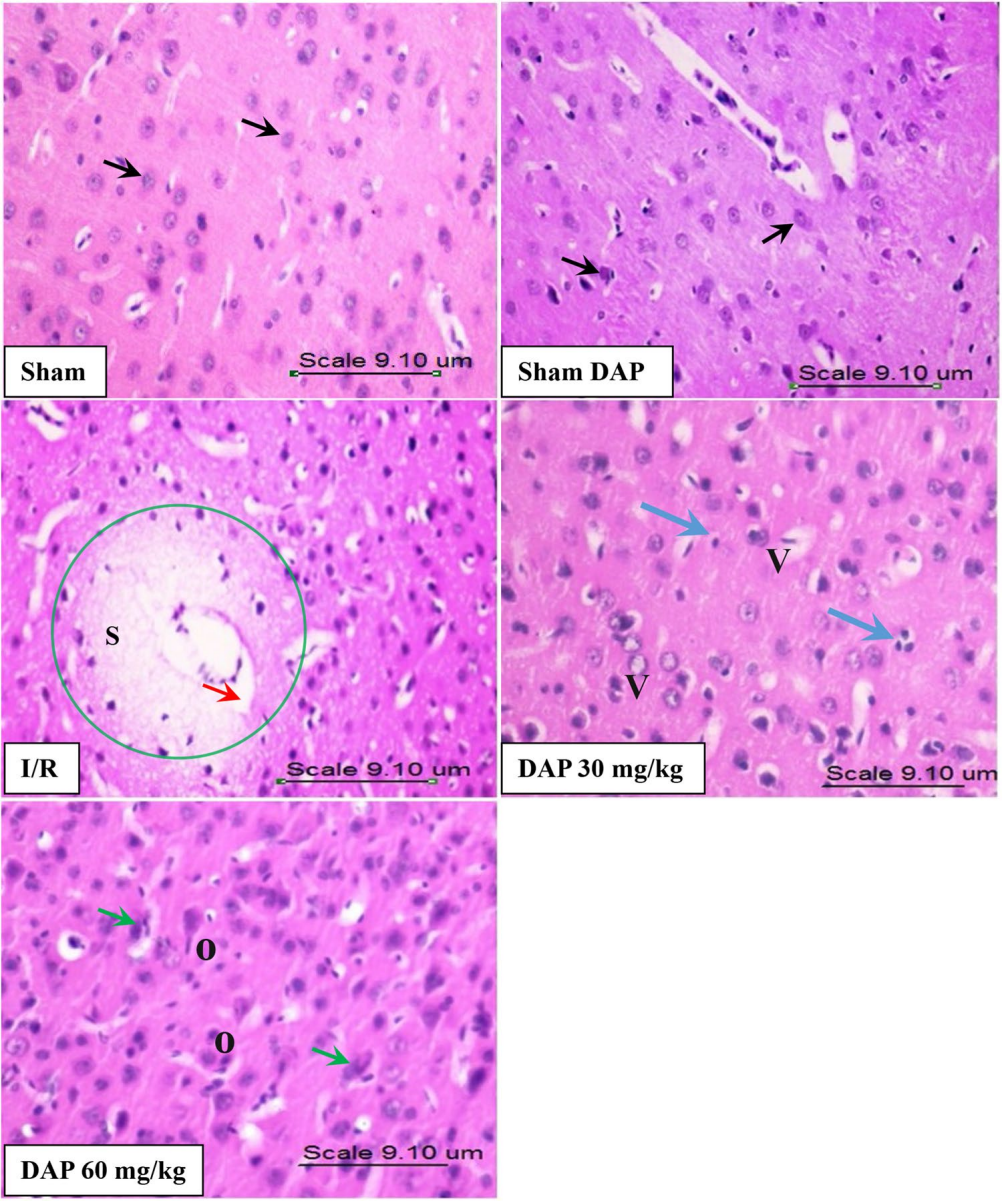
Photomicrographs of the cerebral cortex of the sham-operated group, the sham dapoxetine-pretreated group, the untreated I/R group, the 30 mg/kg dapoxetine–pretreated group, and the 60 mg/kg dapoxetine–pretreated group. Black arrow refers to the normal histological structure of several layers of neuronal cells, containing oval or rounded nuclei surrounded by scanty basophilic cytoplasm, arranged with no sharp boundaries. “S” indicates spongiosis, “V” indicates vesiculated nuclei, and “O” indicates oligodendrocytes. Red arrow refers to perivascular edema, blue arrow refers to microglial cells, and green arrow refers to neurons with shrunken, condensed, and clumped nuclear chromatin. I/R cerebral ischemia/reperfusion

Analysis of H&E-stained tissue sections revealed severe neuronal damage in the cerebral cortex of I/R rats manifested as swollen, atrophic, and shrunken pyknotic nuclei. Furthermore, neurons in these sections appeared with eosinophilic cytoplasm. On the other hand, the same areas in the brains of the sham-operated group showed normally preserved histological structures (Fig. 4 and Table 2). Pretreatment with DAP reduced the histopathological damage induced by ischemic injury. Moreover, the damage score reported in the cerebral cortex of DAP 30 mg/kg–pretreated rats was lower than that of 60 mg/kg dose. Although the high dose of DAP decreased the numbers of apoptotic eosinophilic neurons relative to the I/R group, this effect was more pronounced in animals receiving the small dose.

**Table 2:** Semi-quantitative estimation of the histopathology of brain tissue on global cerebral ischemia/reperfusion and their alteration by different doses of dapoxetine

	Sham	Sham DAP	I/R	I/R + DAP	
				30 mg/kg	60 mg/kg
Cerebral cortex	0	0	** + + + **	** + **	** + + **
Hippocampus	0	0	** + + **	0	** + **

Data are presented as (0) normal, ( +) > 25% of the area is damaged, (+ +) 25–50% of the area is damaged, (+ + +) > 75% of the area is damaged, and (+ +  + +) < 75% of the area is damaged

*I/R* cerebral ischemia/reperfusion, *DAP*: dapoxetine

**Fig. 5:**
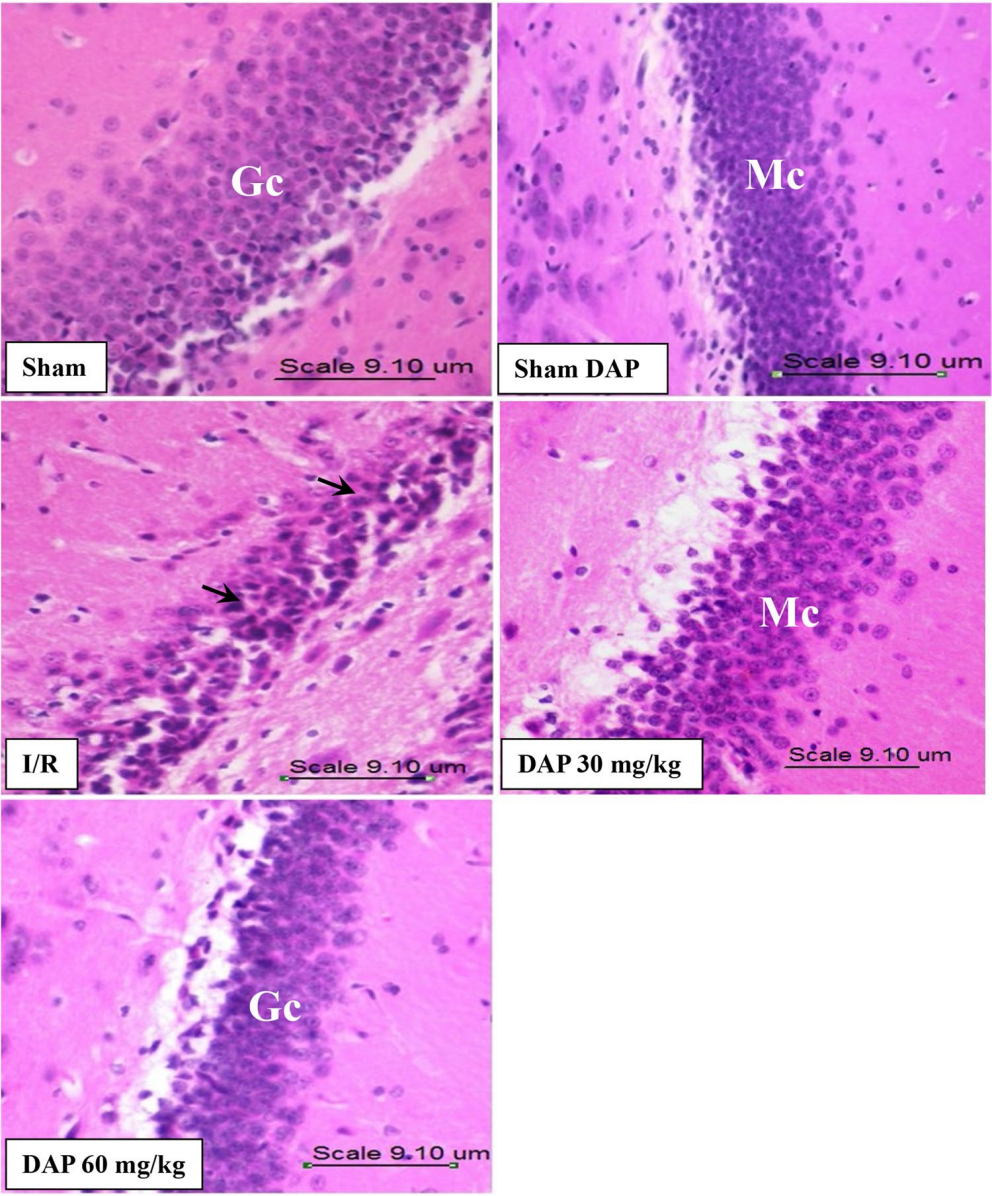
Photomicrographs of the hippocampus of the sham-operated group, the sham dapoxetine-pretreated group, the I/R untreated group, the 30 mg/kg dapoxetine–pretreated group, and the 60 mg/kg dapoxetine–pretreated group. I/R cerebral ischemia/reperfusion, DAP dapoxetine

Data in Fig. 5 and Table 2 showed that I/R injury resulted in hippocampus damage in experimental animals, precisely like it did in the cerebral cortex. The hippocampus of the I/R rats showed disorganized cells, neuronal degeneration, vacuolation, and shrunken, darkly stained, polygonal-shaped nuclei. Importantly, hippocampal sections of these rats showed signs of diffuse neuronal damage in all hippocampal layers compared to sham-operated rats, which showed normal histology. The hippocampal sections of DAP 30 mg/kg–pretreated rats revealed histological features like sham-operated rats, including compact granular cells with dark nuclei. However, sections from the DAP 60 mg/kg–pretreated rats demonstrated cellular disorganization, shrinkage in sizes of pyramidal cells, and darkened nuclei (Fig. 5 and Table 2).

## Discussion

The purpose of this study is to assess the neuroprotective effect of DAP as a short-acting SSRI, on global cerebral ischemia/reperfusion injury. Bilateral common carotid artery occlusion (BCCAO) was employed as a model of transient global cerebral ischemia, which induced consistent damage to different regions of the brain with a low mortality rate (Arabian et al. 2018; Hua et al. 2006).

Our research has shown that BCCAO for 30 min followed by 24 h of reperfusion caused neuronal injury in experimental animals. This injury is confirmed by the noted neurobehavioral alterations as well as by the biochemical and histological data. Our findings, which are in line with earlier studies demonstrated that locomotor and sensorimotor dysfunction is precipitated by the induction of global cerebral ischemia (Chang et al. 2018; M. Li et al. 2016; Shehata et al. 2020). The deteriorated performance scores of I/R-injured rats in the open-field, closed-field, beam-walking, and adhesive-removal tests suggested that I/R injury was successfully inflicted in the current investigation. The massive infarct volumes and significant neuronal damage detected in the brain tissues of these rats, in contrast to the intact tissues collected from the sham-operated counterparts, provided additional proof. The prior administration of DAP remarkably improved the previously mentioned behavioral tests. These findings concur with recent findings, which demonstrated that acute treatment with one of the SSRIs (vortioxetine) displayed a significant efficacy in most behavioral assessments (Sanchez et al. 2015). These findings suggested that DAP has neuroprotective properties against MCAO-induced behavioral and motor impairments.

The brain tissue is vulnerable to oxidative stress because it contains low levels of natural antioxidant enzymes such as superoxide dismutase (SOD), glutathione-peroxidase (GSH-Px), and catalase (CAT), which serve as a cellular defense mechanism against ROS (Jittiwat 2019). MDA, a toxic byproduct of lipid peroxidation, could properly reflect the rate and degree of lipid peroxidation and indirectly reflect the capacity of free radical clearance (Schettler et al. 1999). The outcomes of our investigation accorded with those of earlier studies (Abdel-Latif et al. 2018; Jia et al. 2017; Praveen Kumar et al. 2020). In the current study, DAP pretreatment reduced the increased level in lipid peroxidation caused by I/R injury. It also exerted an antioxidant role after ischemia as it preserved cerebral levels of reduced GSH, but cerebral catalase activity was not noticeably changed. The precise mechanism underlying DAP’s influence on GSH is unknown. The fact that DAP-mediated inhibition of glutamate-induced calcium signaling and mitochondrial depolarization that prevents subsequent excessive formation of reactive oxygen species precludes neuronal cell death, however, is a potential explanation (Jeong et al. 2017). Besides, the overexpression of brain-derived neurotrophic factor, because of SSRI treatment, upregulates various antioxidant enzymes like superoxide dismutase and glutathione-peroxidase after transient global cerebral ischemia (D. H. Kim et al. 2007ab). As a result, optimizing the antioxidant system could be a central focus for novel therapies for ischemic stroke patients.

Reperfusion injury triggers pro-inflammatory mediators through lipid peroxidation, resulting in brain damage (Kalogeris et al. 2014; Kawabori & Yenari 2015). Inflammatory cytokines such as IL-s and TNF-α, among others, play a crucial function as a regulatory factor in the mechanism of inflammation followed by I/R (Hou et al. 2012). In the early stages of cerebral ischemia, an increase in TNF-α release or synthesis is a principal cause of cerebral infarction (Sairanen et al. 2001). The results of this study showed that TNF-α levels in I/R rats were significantly higher than those in the sham group. While, after treatment with DAP, TNF-α levels in brain tissues were notably less than in I/R rats, indicating that the protective effect of DAP on ischemic injury might be mediated by inhibiting inflammatory cytokine production. In agreement with our results, Maes stated that SSRIs have an anti-inflammatory effect by inhibiting the expression of TNF-α in an animal model (Maes 2008). The findings suggested that DAP potentially reduces the signs and symptoms of ischemia damage via modulating inflammatory markers.

Transforming growth factor-beta (TGF-β) is an injury-related peptide. Interestingly, TGF-β is usually undetectable in the normal brain, but it is overexpressed after global and focal ischemia (Vivien & Ali 2006). Different TGF-β isoforms (β1, 2, and 3) and TGF-β receptors (I and II) are upregulated after transient and permanent MCAO (Ata et al. 1999). Our data demonstrated that protein expression of TGF-β RII was upregulated post-I/R injury, whereas DAP could downregulate protein expression of TGF-β RII after ischemic stroke, and this disparity could be attributed to the dose of DAP. On the other hand, it has been reported that TGF-β could ameliorate hippocampal neuronal injury and reduce infarct volume after transient cerebral I/R via an anti-apoptotic mechanism (Q. Wang et al. 2007). These results show the dual effect of TGF-β signaling that can be both protective and detrimental to the brain tissue during I/R injury.

Post-ischemic injury, excessive NO produced as a result of aberrant NOS activation, is hypothesized to be neurotoxic (Wang et al. 2022). Furthermore, NO accelerates oxidative damage by interacting with superoxide anion to form the oxidant molecule peroxynitrite (Beckman et al. 1990). As shown in our experiments, NO levels increased dramatically following reperfusion. It was stated lately that NOS is upregulated, which causes an overload of NO to be released (Bredt 1999). However, our findings indicated that prior DAP treatment involved rescuing the brain from ischemic damage caused by NO production. Moreover, SSRIs such as fluoxetine, sertraline, and fluvoxamine treatment considerably reduced the rise of nitric oxide in the brain caused by LPS (Abdel-Salam et al. 2011). One explanation is that DAP could reduce the NO-mediated generation of free radicals to minimize their destructive effect.

Caspases are potent apoptosis inducers and play key roles in the cytotoxicity caused by ischemia. So caspase-3, the main regulator of apoptosis, was investigated in order to clarify the protective mechanism of DAP (Lee et al. 2007). Typically, the overexpression and activation of the tumor necrosis factor receptor 1 (TNFR1) start the downstream activation of caspases and subsequent programmed cell death during I/R injury (Chen & Goeddel 2002). According to reports, increased caspase-3 activity is a contributor to prolonged neuronal cell death post-cerebral ischemia (N. Wang et al. 2014; Wen et al. 2016). The results of the current work confirmed that cleaved caspase-3 expression in the I/R group was significantly higher than that of the sham group. However, administration of 60 mg/kg DAP significantly inhibited the expression of cleaved caspase-3. Fluoxetine, which belongs to the same pharmacological class as DAP, has been proven to normalize caspase-3 activity against I/R injury, which is the underlying therapy in ischemia injury (F. Xu et al. 2019). The brain cortex and hippocampus’s histopathological alterations and neuronal cell death were dramatically diminished after pretreatment with DAP, indicating that DAP has considerable neuroprotective effectiveness and can mitigate stroke brain injury.

Pretreatment of rats exposed to global I/R injury with DAP at 30 mg/kg resulted in better neurobehavioral and histopathological outcomes than with the 60 mg/kg dose. Although brain tissues of both DAP-treated groups showed similar antioxidant capacities and lipid peroxidation levels, the decline in the cerebral protein expression of inflammatory mediators and caspase-3 was dose dependent. However, the 60 mg/kg DAP–treated rats with lower values for such parameters showed less recovery in major neurobehavioral features after I/R injury. This could be attributed to the dual role that these inflammatory mediators may perform during cerebral ischemia. For example, activation of TNFR2 by TNF-α protects neurons by upregulation of superoxide dismutase (Song et al. 1997), unlike TNFR1 that mediates apoptosis (Chen & Goeddel 2002). Numerous studies showed that functional TNF-α, at certain levels, reduces infarct volume and suppresses ROS-mediated toxicity after I/R, while complete inhibition was deleterious (Shohami et al. 1999). Plus, TNF-α-mediated activation of caspase-3 breaks down PARP and favors apoptotic rather than necrotic cell death (Sairanen et al. 2009). Further support for these findings comes from the work of Raghupathi et al. (1998) who showed that overexpression of Bcl-2 prevented cortical apoptosis in a model of traumatic brain injury. However, Bcl-2 overexpression neither inhibited hippocampal cell death nor inhibited the motor and cognitive deficits (Raghupathi et al. 1998). Moreover, targeted inhibition of caspase-3 did not improve functional outcomes in comparison with the vehicle-treated rats in a similar model, despite a significant reduction in cellular loss (Clark et al. 2000). These previous results, in line with our findings, indicate that finetuned caspase-3 activation, enough to suppress the overactivation of PARP, is essential for neuronal tissue integrity during I/R injury (Raghupathi 2004), which could explain why the 30 mg/kg-treated rats have superior functional outcomes following I/R damage. Taken together, low-grade activation of inflammatory signaling, as well as controlled neuronal apoptosis after ischemia, may be beneficial where the brain can remove injured or damaged cells by apoptosis while minimally affecting the remaining brain tissues, thus avoiding precipitation of diffuse injury.

## Conclusion

Our novel findings establish, for the first time, the ability of DAP to protect against cerebral ischemia/reperfusion injury. The results showed that DAP improved the neurobehavioral outcomes of transient ischemia and attenuated the brain injury induced by I/R. Pretreatment with DAP suppressed the elevation in oxidative stress associated with ischemia and improved the endogenous antioxidant capacity. Besides, DAP attenuated I/R-induced inflammatory and pro-apoptotic responses.

## Data Availability

Datasets used in this work are available upon request.
